# Assessing differential effects of single and accelerated low‐frequency rTMS to the visual cortex on GABA and glutamate concentrations

**DOI:** 10.1002/brb3.1845

**Published:** 2020-09-23

**Authors:** Sara A. Rafique, Jennifer K. E. Steeves

**Affiliations:** ^1^ Department of Psychology and Centre for Vision Research York University Toronto ON Canada

**Keywords:** accelerated rTMS, GABA, glutamate, magnetic resonance spectroscopy, repetitive TMS, visual cortex

## Abstract

**Background:**

The application of repetitive transcranial magnetic stimulation (rTMS) for therapeutic use in visual‐related disorders and its underlying mechanisms in the visual cortex is under‐investigated. Additionally, there is little examination of rTMS adverse effects particularly with regards to visual and cognitive function. Neural plasticity is key in rehabilitation and recovery of function; thus, effective therapeutic strategies must be capable of modulating plasticity. Glutamate and γ‐aminobutyric acid (GABA)‐mediated changes in the balance between excitation and inhibition are prominent features in visual cortical plasticity.

**Objectives and method:**

We investigated the effects of low‐frequency (1 Hz) rTMS to the visual cortex on levels of neurotransmitters GABA and glutamate to determine the therapeutic potential of 1 Hz rTMS for visual‐related disorders. Two rTMS regimes commonly used in clinical applications were investigated: participants received rTMS to the visual cortex either in a single 20‐min session or five accelerated 20‐min sessions (not previously investigated at the visual cortex). Proton (1H) magnetic resonance spectroscopy for in vivo quantification of GABA (assessed via GABA+) and glutamate (assessed via Glx) concentrations was performed pre‐ and post‐rTMS.

**Results:**

GABA+ and Glx concentrations were unaltered following a single session of rTMS to the visual cortex. One day of accelerated rTMS significantly reduced GABA+ concentration for up to 24 hr, with levels returning to baseline by 1‐week post‐rTMS. Basic visual and cognitive function remained largely unchanged.

**Conclusion:**

Accelerated 1 Hz rTMS to the visual cortex has greater potential for approaches targeting plasticity or in cases with altered GABAergic responses in visual disorders. Notably, these results provide preliminary insight into a critical window of plasticity with accelerated rTMS (e.g., 24 hr) in which adjunct therapies may offer better functional outcome. We describe detailed procedures to enable further exploration of these protocols.

## Graphical abstract

We compared the effects of a single session and five accelerated sessions of 1 Hz rTMS to the visual cortex on GABA+ and Glx (glutamate and glutamine) neurotransmitter levels. GABA+ and Glx concentrations were unaltered following a single session of rTMS to the visual cortex. However, accelerated rTMS reduced GABA+ for 24 hr, returning to baseline 1‐week post‐rTMS.

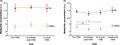

## 1 INTRODUCTION

Transcranial magnetic stimulation (TMS), an evolving noninvasive brain stimulation technique, provides an effective tool to understand and map brain function and connectivity in healthy individuals and patient populations. Repetitive TMS (rTMS; TMS applied repeatedly in a sequence lasting seconds to minutes) is widely used in higher doses for neuromodulatory therapeutic application in a variety of psychological and neurological disorders since it can elicit significant neurophysiological and behavioral after‐effects outlasting stimulation itself (for a review, see Thut & Pascual‐Leone, 2010; Wassermann & Lisanby, 2001). Previous work has demonstrated long‐lasting effects for 1‐year following prefrontal stimulation in depression (Dunner et al., 2014), and improved motor recovery in stroke for at least 6 months following motor cortex stimulation (Liepert, Bauder, Miltner, Taub, & Weiller, 2000). However, mechanisms underlying TMS remain largely unknown due to variability in study methods, mainly with regard to TMS parameters (e.g., stimulation intensity, frequency, and dose), and location of the stimulation site.

Despite the popular use of TMS as a research and therapeutic tool, its potential to modulate neurobiochemical responses in the visual cortex, and hence, its value as a therapeutic technique in visual‐related disorders is under‐investigated. Previously, a valuable therapeutic application of rTMS to the visual cortex has been to modulate chronic and disruptive visual hallucinations that are prevalent following visual pathway damage (Merabet, Kobayashi, Barton, & Pascual‐Leone, 2003; Rafique, Richards, & Steeves, 2016). Other forms of noninvasive brain stimulation (e.g., transcranial direct current stimulation; tDCS) have been used to promote vision restoration in visual field loss (for a review, see Alber, Cardoso, & Nafee, 2015).

Neurobiochemical responses to stimulation drive the overall effect and form the basis of therapeutic intervention (for a review, see Hoogendam, Ramakers, & Di Lazzaro, 2010). The desired neuromodulatory effect largely depends on the stimulation technique and its underlying mechanisms (e.g., rTMS versus tDCS; Stagg, 2014), altered mechanisms associated with the pathophysiology, and the cortical/subcortical structure in question. Glutamate and γ‐aminobutyric acid (GABA), key excitatory and inhibitory neurotransmitters in the mammalian brain, respectively, modulate the induction of long‐term potentiation (LTP) and long‐term depression (LTD) (for a review, see Lüscher & Malenka, 2012). Additionally, glutamine acts as an important precursor in the glutamate/GABA‐glutamine cycle (Bak, Schousboe, & Waagepetersen, 2006). rTMS can exert excitatory or inhibitory cortical effects at the stimulated area in nonvisual cortical regions via glutamate and GABA receptor activity, respectively (Michael et al., 2003; Stagg et al., 2009b; Yue, Xiao‐Lin, & Tao, 2009). Alterations in the delicate homeostatic balance between these neurotransmitters have significant implications in various disorders. For instance, concerning visual disorders, a blockade of GABA leads to an increase in neural firing and is thought to underlie visual hallucinations (Manford & Andermann, 1998). Reduced GABA but unaffected glutamate levels have also been shown to promote visual cortical plasticity and improve visual function in amblyopia (Vetencourt et al., 2008). To advance the clinical application of rTMS for use in visual‐related disorders, we investigated the ability of low‐frequency (1 Hz) rTMS to alter visual cortical GABA and glutamate levels in healthy individuals. Knowledge of rTMS effects in healthy individuals is desirable to determine underlying neurobiochemical mechanisms prior to translation to therapeutic protocols targeted at GABA and glutamate‐mediated responses. This will further aid in determining the predictability of rTMS effects and will minimize adverse effects in pathophysiology. GABA (assessed via GABA+, the combined concentration of GABA and macromolecules) and glutamate (assessed via Glx, the combined concentration of glutamate and glutamine) concentrations were measured using proton (^1^H) magnetic resonance spectroscopy (MRS), a noninvasive tool that enables in vivo quantification of metabolites within a localized region. Metabolites emit a signal of a specific frequency based on their molecular structure, and MRS identifies several metabolites by their discrete peak or a set of peaks in the frequency spectrum (Pfeuffer, Tkáč, Provencher, & Gruetter, 1999). Establishing effects of rTMS to the visual cortex on GABA and glutamate will determine the suitability of its application to visual‐related disorders with known alterations in GABA/glutamate activity or to induce plasticity for recovery of function.

In addition, we compared stimulation protocols since the most efficient stimulation protocol for clinical practice remains under question. In clinical applications, two alternative rTMS regimes are established: a single session or accelerated sessions applied in a single day over consecutive days. Accelerated (also termed within‐session) stimulation consists of multiple sessions within a day and significantly reduces the number of days/weeks of stimulation compared with single session rTMS. We tested a shorter schedule of these two protocols on the visual cortex. The first protocol consisted of a single 20‐min session of low‐frequency (1 Hz) rTMS to the visual cortex. Single sessions of rTMS have been applied over consecutive days for therapeutic application in visual‐related disorders (e.g., Fumal et al., 2006; Rafique et al., 2016) and nonvisual disorders (e.g., Dunner et al., 2014; Liepert, Bauder, et al., 2000; Speer et al., 2000). The second accelerated protocol consisted of five consecutive 20‐min sessions of 1 Hz rTMS to the visual cortex separated by ~15 min in a single day. Advances in the therapeutic use of rTMS in nonvisual disorders demonstrate that accelerated rTMS produces more cumulative, stable, and longer‐lasting effects than single daily sessions over consecutive days/weeks (Goldsworthy, Müller‐Dahlhaus, Ridding, & Ziemann, 2014; Holtzheimer et al., 2010). The effect of accelerated rTMS in visual disorders remains unknown. In addition, accelerated rTMS has better compliance in clinical settings due to the considerably reduced number of visits; for example, five accelerated sessions in a single day are comparable to a single daily session applied over five consecutive days.

A secondary motivation for this study was to continue to refine our previous work where we successfully used rTMS to modulate disruptive visual hallucinations (Rafique et al., 2016; Rafique, Richards, & Steeves, 2018) that prevalently occur following visual pathway damage (Baier et al., 2010; Gordon, 2016). Visual hallucinations are associated with increased vividness of visual imagery and a weak ability to distinguish real perception from mental imagery (Böker, Hijman, Kahn, & Haan, 2000; Mintz & Alpert, 1972). The neural substrates of visual imagery are similar to those of visual perception (Ishai & Sagi, 1995; Kosslyn, Thompson, & Alpert, 1997). Visual imagery critically depends on neural activity in the primary visual cortex and is related to perception and memory that may facilitate cognitive performance (Kosslyn et al., 1999). Therefore, we also assessed the effects of the two alternative low‐frequency rTMS regimes on the vividness of visual imagery and predisposition to visual hallucinations, and their relationship with visual cortical excitability. Understanding the effects of rTMS to visual cortices will provide crucial information to inform recommended therapy in disorders presenting with visual hallucinations (e.g., visual pathway damage, schizophrenia, Parkinson's disease; Burke, 2002; Manford & Andermann, 1998; Yoon et al., 2010).

As far as we are aware, we are the first to investigate accelerated rTMS with a low‐frequency protocol and its application to the visual cortex. To ensure safe and effective use of accelerated rTMS to the visual cortex, we also considered it necessary to assess adverse effects, including effects on basic cognitive function.

## 2 METHODS

This study was approved by York University's Office of Research Ethics. All individuals gave informed written consent.

### 2.1 Participants

Sixteen healthy participants were recruited for the study (mean_age_ ± *SEM* = 25.15 ± 1.21 years; 10 males/6 females). All participants were right‐handed, with normal or corrected‐to‐normal vision (>0.04 logMAR; stereoacuity ≥ 50”), and no known contraindications to TMS and magnetic resonance imaging (MRI). We recruited participants with no known underlying medical conditions, and no history of neurological or psychological disorders. Further, due to interactions with metabolite receptors and/or TMS mechanisms, we recruited participants that were not currently taking any medications (Stell, Brickley, Tang, Farrant, & Mody, 2003) including hormonal contraceptives (Kaore et al., 2012; Smith, Adams, Schmidt, Rubinow, & Wassermann, 2002), with no history of frequent or chronic migraines (Bohotin et al., 2002; Russo et al., 2005), no history of alcohol/substance dependence, and were nonsmokers (Epperson et al., 2005). We additionally asked that participants did not consume alcohol within 48 hr prior to each visit due to potential interactions with metabolites (Lobo & Harris, 2008). Participants received monetary compensation for their participation ($10 CAD/hr).

### 2.2 Experimental design overview

Participants underwent pre‐rTMS (baseline) vision assessments and questionnaires, MRS, and phosphene threshold (PT; visual cortical excitability) measures. In a separate follow‐up visit, participants received offline 1 Hz rTMS to the visual cortex (V1) at the individual PT level, either in a (1) single 20‐min session of rTMS, or (2) five accelerated 20‐min sessions of rTMS (separated by intervals of ~15 min). MRS was repeated immediately following cessation of rTMS in both groups. MRS was further performed (1) 1 hr post‐rTMS in the single rTMS group, and (2) 24 hr and 1‐week post‐rTMS in the accelerated rTMS group. Vision assessments and questionnaires were repeated at follow‐up visits.

### 2.3 Vision assessments, cognitive and imagery questionnaires, and adverse effects

Standard visual testing was conducted to ensure normal visual status. Monocular visual acuity was measured using the standardized ETDRS vision chart (Precision Vision, La Salle, IL), stereoacuity was measured using the Titmus circles test (Stereo Optical Company Inc., Chicago, IL), and color vision was assessed using the Ishihara test (Kanehara Trading Inc., Tokyo, Japan). Ocular muscle status involved assessment with the cover test (distance and near) and near point of convergence.

To ensure normal cognitive status, we administered the Montreal Cognitive Assessment (MoCA, v7.1‐7.3), a brief 30‐point cognitive screening test to detect mild cognitive impairment (Nasreddine et al., 2005). MoCA evaluates attention, concentration, working memory, short‐term memory, delayed recall, language, visuospatial, orientation, and executive function. A different version of the MoCA was administered at each follow‐up visit.

Vividness of visual imagery was measured using the Vividness of Visual Imagery Questionnaire (VVIQ), a 16‐item questionnaire (Marks, 1973). VVIQ was performed initially with eyes open and then repeated with eyes closed. We reversed the 5‐point rating scale, so that 1 = *no image at all, you only know that you are thinking of an object*, and 5 = *perfectly clear and as vivid as normal vision*, which provides a more intuitive scale.

Unusual positive perceptual experiences were assessed using a revised Launay‐Slade Hallucination Scale (LSHS), a 16‐item questionnaire. The LSHS measures predispositions to hallucinations in healthy and clinical populations (Launay & Slade, 1981, revised by Morrison, Wells, & Nothard, 2000). Questions were rated on a 4‐point scale to measure the frequency of the hallucinatory event (1 = *never*, 2 = *sometimes*, 3 = *often*, 4 = *almost always*).

Further, we asked participants to report changes in mood, concentration, cognition, sensory disruption (e.g., visual or hearing changes), as well as headaches, neck pain, or any other symptoms following rTMS. Adverse effects were reported on a 5‐point scale (1 = *no symptoms/change*, 2 = *minimal symptoms/change*, 3 = *slight symptoms/change*, 4 = *moderate symptoms/change*, 5 = *significant symptoms/change*).

We repeated all vision assessments and questionnaires at follow‐up visits to assess any changes with rTMS, and for a more complete representation of longer‐term effects of 1 Hz rTMS to the visual cortex.

### 2.4 Magnetic resonance imaging acquisition

Anatomical and ^1^H MRS data were acquired with a 3T Siemens Magnetom^®^ Tim Trio magnetic resonance scanner, with a 32‐channel high‐resolution brain array coil (Siemens, Erlangen, Germany). Head motion was minimized with the placement of soft pads holding the participant's head in place. Imaging was acquired at rest in a dark room, and participants were instructed to keep their eyes closed throughout.

Anatomical images were acquired first to allow placement of the MRS volume‐of‐interest (VOI) using a T1‐weighted magnetization‐prepared rapid gradient echo (MPRAGE) imaging sequence (number of slices = 192; in‐plane resolution = 1 × 1 mm; slice thickness = 1.0 mm; imaging matrix = 256 × 256; repetition time (TR) = 2,300 ms; echo time (TE) = 2.62 ms; inversion time = 900 ms; flip angle = 9°; field of view (FoV) = 256 mm; acquisition time = ~5 min).

**Figure 1:**
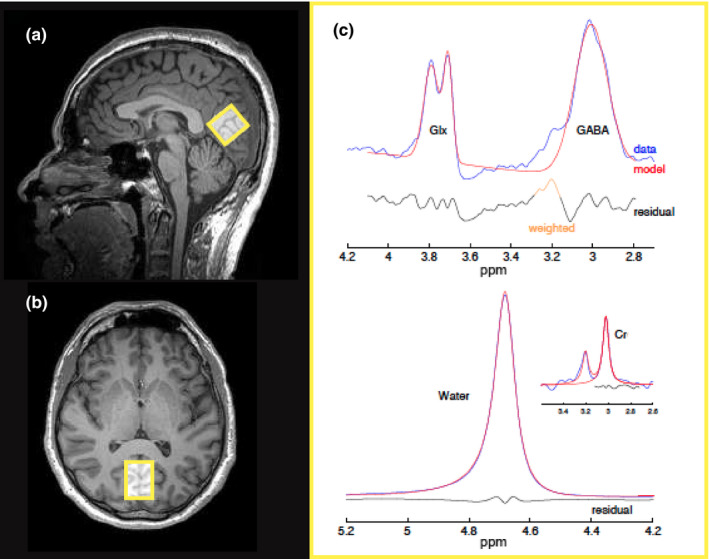
Illustration of proton (^1^H) MR spectra acquired from the visual cortex. (a, b) Example of typical magnetic resonance (MR) spectroscopy volume‐of‐interest (VOI) placement in the occipital lobe on T1‐weighted images for a single participant shown in sagittal and transverse planes, respectively. (c) Sample processing of the MEGA‐PRESS difference edited spectrum (blue line) from the corresponding VOI using Gaussian fitting (red line) with Gannet (v3.0). Example shows typical signal peaks for GABA (3.02 ppm), Glx (glutamate and glutamine composite; 3.75–3.8 ppm), creatine (Cr; 3.0 ppm), and water (4.7 ppm) for a single participant. ppm = parts per million

Using the anatomical images, MRS was acquired from a single 2.5 × 2.5 × 2.5 cm^3^ VOI positioned medially in the visual cortex (V1). The VOI was positioned as far back within the posterior region of the occipital pole, centered on the calcarine sulcus, and avoided nonbrain tissue (including cerebrospinal fluid [CSF] and the sagittal sinus) to minimize macromolecule contamination. The lower edge followed the cortical surface, and was aligned alongside the cerebellar tentorium (Figure 1a,b). The VOI position was recorded relative to anatomical landmarks in all three dimensions (sagittal, coronal, and transverse) using screenshots, and used as a reference for subsequent acquisitions. In two participants, because of a relatively small occipital lobe, the VOI had to be placed further supero‐anteriorly, and may not have been constrained fully to the occipital lobe. The use of a smaller VOI was not desirable since it would reduce the signal‐to‐noise ratio (for a review, see Mullins et al., 2014). ^1^H MR spectra were obtained using Mescher‐Garwood point resolved spectroscopy (MEGA‐PRESS), a J‐coupled difference editing technique (Mescher, Merkle, Kirsch, Garwood, & Gruetter, 1998), provided by Siemens as a work‐in‐progress (WIP) sequence (TR = 1,500 ms; TE = 68 ms; spectral bandwidth = 1,500 Hz; 1,024 data points with water suppression yielding 512 averages; acquisition time = ~13 min). The MEGA‐PRESS sequence provides reliable detection of GABA and other brain metabolites (Mullins et al., 2014; Puts & Edden, 2012). Siemens standard three‐dimensional (3D) automated shimming, followed by manual shimming was performed before each acquisition. During odd‐numbered (“ON resonance”) acquisitions, a frequency selective Gaussian inversion pulse, and a chemical shift selective suppression (CHESS) water suppression band at 4.7 parts per million (ppm) of proton frequency were irradiated at 1.9 ppm. During even‐numbered (“OFF resonance”) acquisitions, the same pulse was applied symmetrically to the opposite side of the water spectrum at 7.5 ppm. The difference between the “ON” and “OFF” edited spectra provides a resulting spectrum retaining only peaks affected by the editing pulses: the GABA signal peak at 3.02 ppm, a combined glutamate and glutamine (Glx) signal peak at 3.75–3.8 ppm, and macromolecular peaks. The difference editing approach separates the GABA spectrum from overlapping spectra of more concentrated metabolites, in particular the creatine (Cr; an amino acid) peak at 3.0 ppm (Figure 1c, blue line). However, the GABA spectrum still contains contributions from other macromolecules (Aufhaus et al., 2013; Behar, Rothman, Spencer, & Petroff, 1994) and homocarnosine (a GABA derivative inhibitory neuromodulator; Rothman, Behar, Prichard, & Petroff, 1997), and is therefore referred to as GABA+ rather than GABA. Due to their similar chemical structure and overlapping spectra, glutamate (3.75 ppm) and its metabolic intermediate glutamine (3.76 ppm) cannot be differentiated using this method and therefore are referred to as the composite measure Glx. An unsuppressed water reference was also acquired (16 averages, acquisition time = ~1 min).

### 2.5 Transcranial magnetic stimulation

A Magstim Rapid^2^ Stimulator and a 70‐mm‐diameter figure‐of‐eight coil (Magstim, Whitland, Wales, UK) were used to deliver stimulation pulses to the defined stimulation site.

#### 2.5.1 Phosphene threshold

Due to large interindividual variability in visual cortical excitability thresholds (Stewart, Walsh, & Rothwell, 2001), rTMS was delivered to the target stimulation site at the individual PT level to minimize potential confounds of individual differences in excitability and, therefore, induce consistent effects across participants. PT provides a well‐established standard measure of visual cortical excitability (Silvanto & Pascual‐Leone, 2008). PT was measured using 1 Hz rTMS (7‐s interstimulus interval, 10 pulses) to the occipital lobe, and defined as the lowest stimulator output intensity (expressed as a percentage of maximal output) that evoked phosphenes in at least 50% of pulses (Elkin‐Frankston, Fried, Pascual‐Leone, Rushmore, & Valero‐Cabré, 2010). Participants were seated in a lit room wearing a blindfold with eyes closed, and asked to report “yes/no/maybe” to phosphene elicitation following each TMS pulse. TMS was initially applied 2 cm lateral to the inion, at an initial stimulator output intensity of 60%. The coil handle was held 90° to the left of the inion midline. If the participant reported a visual sensation, they were asked to describe the appearance (color, shape, and size) and location to confirm phosphene perception. A “maybe” response was considered a negative response. If no visual sensation was reported, the TMS coil was repositioned in 1 cm steps above/below/lateral to the original site 2 cm lateral to the inion, and pulses were re‐delivered, to a maximum of 4 cm lateral and above/below the inion. If phosphenes were still not reported, the stimulator output intensity was increased by 5% and the process repeated. The process was repeated until a phosphene was perceived, and the stimulator output intensity was then refined in 1% steps. Blindfolds were removed every 10–15 min where necessary, for a minimum of 2 min, to prevent visual cortical excitability from dark adaptation (Boroojerdi, Bushara, et al., 2000).

#### 2.5.2 Repetitive transcranial magnetic stimulation

Participants' anatomical MR images were reconstructed to 3D cortical surfaces using Brainsight software (Rogue Research, Montreal, QC, Canada). The stimulation site corresponded to the centre of the MRS VOI (mean_depth_ ± *SEM* = 38.631 ± 0.74 mm). The stimulation site was mapped on each participant's corresponding anatomical images in Brainsight by matching anatomical landmarks to the MRS VOI screenshots obtained at pre‐rTMS MRS acquisition. The spatial relationship between the stimulation site and reference points from the participant's head (tip of nose, nasion, right, and left tragus) were co‐registered using a Polaris infrared tracking system (Northern Digital Instruments, Kitchener, ON, Canada). Movement of the coil with respect to the participant's head, and therefore the stimulation site, was visualized in real‐time using the infrared image‐guided stereotaxy to ensure accurate positioning of the coil and targeted disruption of the stimulation site throughout rTMS. The TMS coil was held tangential to the surface of the skull to minimize coil‐to‐cortex distance and to maximize the TMS effect (Ulmer & Jansen, 2010). Participants were seated in a comfortable position with an adjustable chin rest to limit head movement and provided with earplugs to prevent changes in auditory thresholds during rTMS (Rossi, Hallett, Rossini, & Pascual‐Leone, 2009). We used a parallel group design. Participants underwent 1 Hz rTMS (1 s interstimulus interval, 1,200 pulses [20 min], 100% PT) at rest, either in a single 20‐min session (*n* = 8, 4 males/4 females) or five accelerated 20‐min sessions of the same protocol separated by ~15 min in a single day (*n* = 8, 6 males/2 females). Intervals of 10–20 min in accelerated stimulation are considered to produce longer‐lasting effects (for a review, see Goldsworthy, Pitcher, & Ridding, 2015) compared to shorter intervals, for example, 3 min (Monte‐Silva, Kuo, Liebetanz, Paulus, & Nitsche, 2010) or 5 min (Bastani & Jaberzadeh, 2014). Our chosen neuromodulation regime was further based on evidence that 1 Hz rTMS to the visual cortex induces dishabituation of electrophysiological responses (visual evoked potentials), whereas 10 Hz (high‐frequency) rTMS of comparable pulses has no significant effect (Bohotin et al., 2002; Fumal et al., 2003). Moreover, the application of a single daily 15‐min session of 1 Hz rTMS to the visual cortex for 5 consecutive days produces an accumulative effect in dishabituation (Fumal et al., 2006). We have previously obtained an accumulative effect in modulating visual hallucinations following visual pathway damage with a 30‐min session of 1 Hz rTMS to the visual cortex for 5 consecutive days (Rafique et al., 2016). We considered five consecutive 30‐min sessions taxing on participants in the accelerated protocol; thus, in the present study, we opted for a minimum of 20 min of stimulation since it is more effective than shorter application times and reduces interindividual variability (Aydin‐Abidin, Moliadze, Eysel, & Funke, 2006).

### 2.6 Experimental procedure

To minimize the diurnal variation of neuromodulators, including those implicated in TMS mechanisms (e.g., Ridding & Ziemann, 2010; Sale, Ridding, & Nordstrom, 2007), participants underwent testing at approximately the same time of day for all visits. We attempted to perform rTMS as close as possible to the time at which PT was obtained while allowing for MRI acquisition of different visits (pre‐ and post‐rTMS) to be acquired at similar times irrespective of group allocation. Visit 1 (baseline/pre‐rTMS): all participants initially underwent vision assessments and questionnaires to ensure inclusion criteria were met (including normal visual and cognitive status), followed by MRI, which commenced at ~13:00. PT was always obtained after pre‐rTMS MRI, usually on the same day, or at a similar time on a different day. Visit 2 (rTMS): offline 1 Hz rTMS was scheduled at least 4 days following pre‐rTMS measures in both groups to prevent any lingering TMS effects from PT measurement interacting with the rTMS protocol. rTMS commenced at ~13:40 for participants in the single rTMS session group, and at ~11:00 for participants in the accelerated rTMS sessions group. Participants were immediately transferred into the MRI scanner after rTMS ceased, and MRI acquisition commenced within 5 min of rTMS ending. In both groups, immediate post‐rTMS MRI commenced at ~14:00. Participants in the single rTMS group underwent a further MRI 1 hr post‐rTMS at ~15:00. We did not perform further follow‐up visits in the single rTMS group based on previous research demonstrating that aftereffects following ~15–20 min 1 Hz rTMS to the occipital cortex recover within 20–40 min (for a review, see Thut & Pascual‐Leone, 2010). We did not perform MRI 1 hr post‐rTMS in the accelerated rTMS group because of fatigue following a long protocol (~5 hr), and since effects were expected to persist for >24 hr (for a review, see Goldsworthy et al., 2015). Visits 3 and 4 (follow‐up for the accelerated rTMS group only): participants in the accelerated rTMS group were further followed up at 24 hr, and 1‐week post‐rTMS, in both cases at ~14:00.

**Figure 2:**
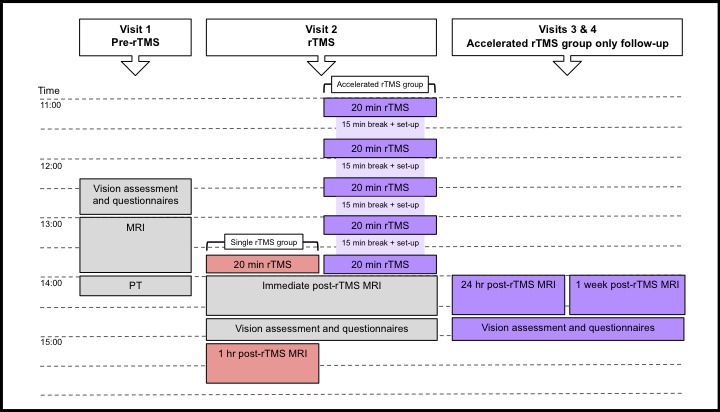
Diagram of the experimental procedure. All participants took part in procedures in gray; participants undergoing a single rTMS session took part in procedures in red; and participants who underwent accelerated rTMS took part in procedures in purple. Pre‐rTMS (baseline) procedures took longer than follow‐up visits as participants were provided with detailed instruction of procedures, and because of time involved in determining optimal positioning of the magnetic resonance spectroscopy volume‐of‐interest. MRI, magnetic resonance imaging; PT, phosphene threshold; rTMS, repetitive transcranial magnetic stimulation

MRI, vision assessments, and questionnaires were repeated at follow‐up visits. Because participants were immediately transferred to the MRI following rTMS cessation, we could not perform vision assessments and questionnaires immediately post‐rTMS in both groups; instead, they were performed after immediate post‐rTMS MRI acquisition. Therefore, for the single rTMS group, vision assessments and questionnaires were only followed up once. Figure 2 shows a diagram of the procedure for both rTMS groups.

### 2.7 Data analyses

#### 2.7.1 Magnetic resonance spectroscopy processing

MEGA‐PRESS data were exported in RDA file format. Data were processed using the MATLAB‐based tool Gannet (v3.0; http://www.gabamrs.com; Edden, Puts, Harris, Barker, & Evans, 2014). To filter out spectra of interest and improve the signal‐to‐noise ratio, individual acquisitions underwent standard processing including frequency and phase correction, fast Fourier transform of time‐domain acquired data to frequency‐domain spectra, exponential line broadening, subtraction of the “ON” and “OFF” spectra resulting in the difference edited spectrum, and Gaussian model fitting of the GABA+ and Glx signal peaks. The amplitude of the peak for a given metabolite is related to the total number of molecules and therefore represents the total concentration of that metabolite. GannetFit estimated the area under the edited GABA+, Glx, and Cr peaks, and the water signal from the unsuppressed water spectrum (Figure 1c). GannetCoRegister, which calls SPM8 (Statistical Parametric Mapping, Wellcome Centre for Human Neuroimaging, London, UK; http://www.fil.ion.ucl.ac.uk/spm/), registered the MRS VOIs to the corresponding anatomical images by creating a binary mask of the VOI with the same geometric parameters as the anatomical image. GannetSegment performed segmentation of the anatomical images, and determined tissue fractions (gray and white matter, and CSF) within the VOI to provide CSF‐corrected GABA+ and Glx concentration estimates (in institutional units [i.u.]) using SPM8. Lastly, GannetQuantify accounted for tissue‐related variance to provide tissue‐corrected (relaxation‐ and alpha‐corrected, voxel‐average‐normalized) GABA+ and Glx concentration estimates relative to water (i.u.) (Harris, Puts, & Edden, 2015). Gannet uses the standard deviation of the fitting residual, expressed as a percentage of the signal height, to determine the fit error of the model, which reflects the signal‐to‐noise ratio. All acquired spectra had a fit error < 5%.

#### 2.7.2 Statistical analyses

All statistical analyses were performed using R statistical computing software (R Foundation for Statistical Computing, Vienna, Austria; www.R‐project.org). Data were assessed for statistical assumptions and found to violate assumptions of parametric testing. We used multilevel (mixed) modeling and the maximum‐likelihood estimation to analyze differences across multiple variables since it accounts for the repeated nature of the data, is highly flexible in dealing with varying intervals between measurements, and overcomes issues where assumptions of analysis of variance (ANOVA) are violated. Akaike's information criterion was used to measure the goodness of a fit of an estimated model, and the appropriate covariance structure. Where only two variables were compared, we conducted independent/dependent Yuen's *t* tests (*YW*) for non‐normally distributed data with 10% trimmed means. Effect sizes for Yuen (ES*_YW_*) were calculated using 10% trimmed means, and additionally 500 bootstrapping samples for independent *t* tests. Significance level was chosen as *p *< .05, and *p *< .1 for trending results, for all analyses. The rTMS groups were analyzed separately due to different follow‐up visit intervals (single rTMS group: pre‐rTMS, immediate post‐rTMS, 1 hr post‐rTMS; accelerated rTMS group: pre‐rTMS, immediate post‐rTMS, 24 hr post‐rTMS, 1‐week post‐rTMS).

To ensure consistent VOI positioning in participants across visits, we assessed changes in tissue proportions within the VOI between visits for each rTMS group using a multilevel model, with random effect for participant, and fixed effect for visit, for each tissue fraction (gray matter, white matter, CSF). The rTMS groups were analyzed separately due to different follow‐up visit intervals (single rTMS group: pre‐rTMS, immediate post‐rTMS, 1 hr post‐rTMS; accelerated rTMS group: pre‐rTMS, immediate post‐rTMS, 24 hr post‐rTMS, 1‐week post‐rTMS). To ensure tissue proportions were consistent between rTMS groups, pre‐rTMS tissue fractions were compared using Yuen's independent *t* test.

The impact of single and accelerated rTMS sessions on GABA+ and Glx concentrations were analyzed using a multilevel model, with random effect for participant and fixed effect for visits, for each metabolite and rTMS group separately. The effects of rTMS on all vision assessments and questionnaire responses (MoCA, VVIQ, and LSHS) were determined using Yuen's dependent *t* test for the single rTMS group (pre‐rTMS and immediate post‐rTMS visits); and a multilevel model for the accelerated rTMS group (all visits), with random effect for participant and fixed effect for visits.

The relationship between PT (visual cortical excitability), pre‐rTMS GABA+ and Glx concentrations, and pre‐rTMS questionnaire responses was analyzed using Kendall's tau (*τ*), a nonparametric correlation for non‐normally distributed data.

## 3 RESULTS

### 3.1 Tissue fraction analyses

Analyses of tissue fractions demonstrated consistent VOI positioning across visits. In the single rTMS group, there were no significant changes in tissue proportions across visits for white matter, *F*(2, 14) = 2.61, *p *= .109; and CSF, *F*(2, 14) = 1.839, *p *= .195. Tissue proportions for gray matter trended significance across visits, *F*(2, 14) = 3.147, *p *= .074.

In the accelerated rTMS group, there were no significant changes in tissue proportions across visits for gray matter, *F*(3, 18) = 0.719, *p *= .554; white matter, *F*(3, 18) = 0.529, *p *= .668; and CSF, *F*(3, 18) = 0.747, *p *= .538.

Based on pre‐rTMS tissue proportions, there were no significant differences between the single and accelerated rTMS groups in gray matter, *YW*(10.76) = 0.764, *p *= .462, ES*_YW_* = 0.322; white matter, *YW*(10.9) = 0.442, *p *= .668, ES*_YW_ *= 0.223; and CSF, *YW*(9.72)* *= 0.519, *p *= .615, ES*_YW_* = 0.189.

### 3.2 Effect of 1 Hz repetitive transcranial magnetic stimulation on GABA+ and Glx concentrations

In the single rTMS group, there were no significant changes across visits in GABA+ concentration, *F*(2, 14)* *= 0.733, *p *= .498; and Glx concentration, *F*(2, 14)* *= 0.15, *p *= .862.

In the accelerated rTMS group, there was a significant change in GABA+ concentration across visits, *F*(3, 21) = 5.446, *p *= .006. Pre‐rTMS GABA+ concentration was significantly greater than immediate post‐rTMS, *b *= −0.285, *t*(21) = −2.203, *p *= .039; and showed a trend toward being significantly greater than 24 hr post‐rTMS concentration, *b* = −0.229, *t*(21) = −1.768, *p* = .092. Pre‐rTMS GABA+ concentration was comparable with 1‐week post‐rTMS concentration, *b* = 0.178, *t*(21) = 1.372, *p* = .185. There were no significant changes across visits for Glx concentration, *F*(3, 21) = 0.81, *p* = .503.

**Figure 3:**
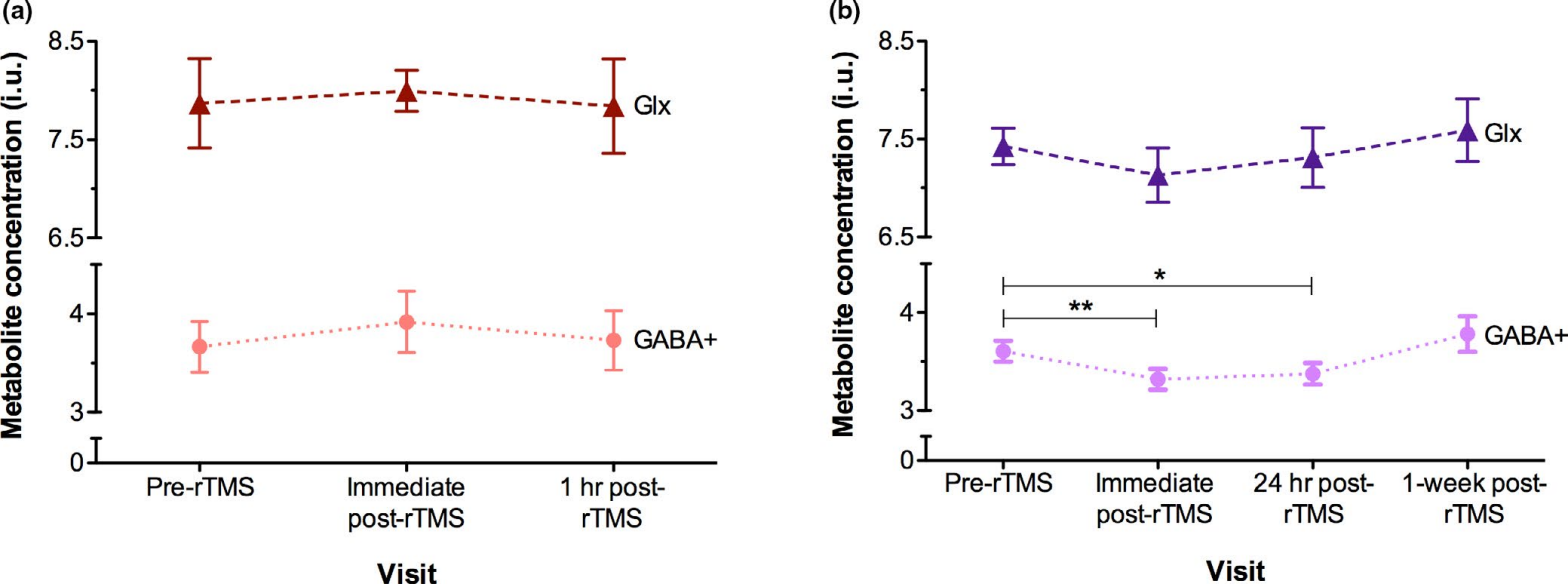
Mean visual cortical GABA+ and Glx concentrations following 1 Hz rTMS. Metabolite concentrations at the visual cortex following (a) a single 20‐min 1 Hz rTMS session, and (b) five accelerated 20‐min 1 Hz rTMS sessions. Pre‐rTMS GABA+ concentrations in the accelerated rTMS group were significantly greater than immediately post‐rTMS (**p < .05), and trended significance with 24 hr post‐rTMS (*p < .1). Error bars represent ± *SEM*. Triangle symbols represent Glx, and circles represent GABA+ concentrations. GABA+, GABA and macromolecules composite; Glx, glutamate and glutamine composite; i.u., institutional units; rTMS, repetitive transcranial magnetic stimulation

Results for metabolite concentrations measured at each visit are presented in Figure 3.

### 3.3 Effect of 1 Hz repetitive transcranial magnetic stimulation on vision assessments, cognitive, and imagery questionnaire responses

For vision assessments, in the single rTMS group, there were no significant differences across visits for right eye visual acuity, *YW*(7) = −0.424, *p* = .685, ES*_YW_* = 0.009; left eye visual acuity, *YW*(7) = −1.286, *p* = .901, ES*_YW_* = 0.002; and stereoacuity, *YW*(7) = 1, *p* = .351, ES*_YW_* = 0.11. In the accelerated rTMS group, there were no significant differences across visits for right eye visual acuity, *F*(3, 21) = 1.629, *p* = .213; left eye visual acuity, *F*(3, 21) = 0.597, *p* = .624; and stereoacuity, *F*(3, 21) = 1.355, *p* = .284. In both rTMS groups, participants correctly identified all color vision plates at all visits.

For questionnaire responses, in the single rTMS group, VVIQ with eyes closed was significantly greater at pre‐rTMS compared with 1 hr post‐rTMS, *YW*(7) = 2.481, *p* = .042, ES*_YW_* = 0.37. There were no significant differences in the single rTMS group across visits for VVIQ with eyes open, *YW*(7) = 0, *p* = 1, ES*_YW_* = 0; MoCA, *YW*(7) = −0.475, *p* = .649, ES*_YW_* = 0.14; and LSHS, *YW*(7) = 1.764, *p* = .121, ES*_YW_* = 0.16. In the accelerated rTMS group, there was a significant difference in MoCA across visits, *F*(3, 21) = 3.117, *p* = .048; contrasts revealed only a trend toward significance with a lower score pre‐rTMS compared to 1‐week post‐rTMS, *b* = 0.875, *t*(21) = 1.987, *p* = .06. There were no significant differences in the accelerated rTMS group across visits for VVIQ with eyes open, *F*(3, 21) = 0.944, *p* = .437; VVIQ with eyes closed, *F*(3, 21) = 0.232, *p* = .873; and LSHS, *F*(3, 21) = 0.684, *p* = .572. Mean (±*SEM*) questionnaire measures are reported in Table 1. Participants' self‐reported symptoms following rTMS are reported in Table 2.

**Table 1:** rTMS group characteristics and questionnaire responses across visits

Visit	Age (years)	PT (%)	MoCA	LSHS	VVIQ	
					eyes open	eyes closed
Single rTMS group	25.5 ± 2.25	68.625 ± 2.05				
Pre‐rTMS			28.875 ± 0.55	22.38 ± 1.349	61.88 ± 3.34	65.875 ± 3.22
1 hr post‐rTMS			29.13 ± 0.295	21.38 ± 1.36	61.88 ± 3.303	59.625 ± 4.63
Accelerated rTMS group	24.75 ± 1.07	73.13 ± 2.75				
Pre‐rTMS			28.875 ± 0.52	21.63 ± 1.511	52.875 ± 2.955	54.625 ± 2.6
1 hr post‐rTMS			29 ± 0.423	4.93 ± 1.742	50 ± 3.059	53.13 ± 4.147
24 hr post‐rTMS			28.875 ± 0.61	22.875 ± 2.06	50.875 ± 3.55	54.429 ± 4.38
1‐week post‐rTMS			29.75 ± 0.16	22.875 ± 1.87	50.25 ± 3.56	52.75 ± 3.9

Note

The columns list (left to right) rTMS group (and visits for which measures are associated with), group age, PT, and questionnaire responses. Data are presented as mean (±*SEM*) values.

Abbreviation: LSHS, revised Launay‐Slade Hallucination Scale; MoCA, Montreal Cognitive Assessment; PT, phosphene threshold; rTMS, repetitive transcranial magnetic stimulation; VVIQ, Vividness of Visual Imagery Questionnaire.

**Table 2:** Adverse effects reported at post‐rTMS follow‐up visits

rTMS group/Participant #	Visit	
	1 hr post‐rTMS	24 hr post‐rTMS
Single rTMS group		
2	Headache = 3	
3	Cognition = 2	
7	Headache = 2	
8	Concentration = 2	
Accelerated rTMS group		
10	Headache = 2	Headache = 4
11	Headache = 2	
12	Neck discomfort = 2	
13	Concentration = 3.5, hearing TMS tapping noises = 3	Hearing TMS tapping noises = 2

Note

The columns list (left to right) participants (identified numerically) that experienced adverse effects following rTMS and their allocated rTMS group, and the post‐rTMS follow‐up visit at which symptoms were experienced. Symptoms were reported as: 1 = *no symptoms/change*, 2 = *minimal symptoms/change*, 3 = *slight symptoms/change*, 4 = *moderate symptoms/change*, 5 = *significant symptoms/change*. Participants reported no significant adverse effects (e.g., pain/discomfort) during or after rTMS, even at higher stimulation intensities. The side effects reported are transient and common with rTMS (Rossi et al., 2009).

### 3.4 Relationship between visual cortical excitability, GABA+ and Glx, and questionnaire responses

There was a significant positive correlation between pre‐rTMS GABA+ and Glx concentrations, *τ* = 0.68, *p *< .001. PT was not significantly correlated with pre‐rTMS measures of GABA+ concentration, *τ* = −0.237, *p* = .206; Glx concentration, *τ* = −0.245, *p* = .19; VVIQ with eyes open, *τ* = −0.254, *p* = .175; VVIQ with eyes closed, *τ* = −0.017, *p* = .928; or LSHS, *τ* = −0.309, *p* = .109. Pre‐rTMS GABA+ concentration was not significantly correlated with corresponding visit measures of VVIQ with eyes open, *τ* = −0.067, *p* = .718; VVIQ with eyes closed, *τ* = −0.136, *p* = .47; or LSHS, *τ* = 0.219, *p* = .254. Similarly, pre‐rTMS Glx concentration was not significantly correlated with corresponding visit measures of VVIQ with eyes open, *τ* = −0.092, *p* = .62; VVIQ with eyes closed, *τ* = −0.093, *p* = .619; or LSHS, *τ* = 0.174, *p* = .362. Mean (±*SEM*) PT and questionnaire responses are reported in Table 1.

## 4 DISCUSSION

We investigated the effects of accelerated rTMS to the visual cortex, which is previously unknown; and provide a method of comparing effects to more traditional protocols (e.g., single session rTMS). We describe detailed methods and procedures to enable further work on this approach in visual‐related disorders.

We found that a single 20‐min session of low‐frequency (1 Hz) rTMS (100% PT) to the visual cortex had no significant effect on GABA+ or Glx concentrations compared to pre‐rTMS levels. Therefore, 20 min of 1 Hz rTMS can be used without modifying GABA+ or Glx concentrations at V1. This may be essential in settings where it is desirable to control for neuroplasticity. However, five accelerated sessions using the same stimulation parameters significantly reduced GABA+ concentration immediately following rTMS, with trending effects lasting up to 24 hr, and returning to baseline by 1‐week post‐rTMS. Despite a positive relationship between pre‐rTMS GABA+ and Glx concentrations, there was no significant change in Glx concentration following accelerated rTMS compared to pre‐rTMS levels, highlighting the specific differential effect of low‐frequency rTMS.

### 4.1 Effect of 1 Hz repetitive transcranial magnetic stimulation on visual cortical GABA+ and Glx concentrations

According to traditional views on frequency‐dependent rTMS effects, we might expect an “inhibitory” response with a low‐frequency (e.g., 1 Hz) rTMS protocol (for reviews, see Hallett, 2007; Thut & Pascual‐Leone, 2010) and accordingly an increase in GABA+ concentration following rTMS. We observed no change in GABA+ concentration following a single session of 1 Hz rTMS, and instead a *decrease* in GABA+ concentration following accelerated 1 Hz rTMS. Our results are also in line with our previous patient study in which we used low‐frequency rTMS to ameliorate visual hallucinations that occurred as a consequence of occipital stroke (Rafique et al., 2016). Although the rTMS protocol in our previous patient study used the classic multiday approach to rTMS, in which the patient underwent a single daily session of 1 Hz rTMS to the visual cortex over 5 consecutive days, we observed an *increase* in functional activity at the stimulation site post‐rTMS (and a corresponding decrease in hallucinations). Following the notion that low‐frequency rTMS is simply inhibitory, we would have expected a decrease in functional activity at the stimulation site in the patient. A number of studies demonstrate that in addition to the frequency, the intensity level relative to the excitability threshold (e.g., motor threshold [MT]) influences whether stimulation induces excitation or inhibition in the cortex. In the motor cortex, sub‐MT paired‐pulse TMS suppresses motor cortex excitability by activating intracortical inhibitory circuits, while supra‐MT intensity facilitates a response (Nakamura, Kitagawa, Kawaguchi, & Tsuji, 1997). Conversely, supra‐MT low‐frequency rTMS to the motor cortex does suppress motor evoked potentials (Chen et al., 1997). High doses of short trains of 1 Hz rTMS to the prefrontal cortex excite local and contralateral functional activity and show intensity‐dependent increases with higher intensities, e g., ≥ 100% MT (Nahas et al., 2001). Therefore, sub‐PT intensity rTMS in our study may have had a suppressive effect on GABA+ concentration. Similar intensity‐dependent rTMS effects are also observed in regional cerebral blood flow. Low‐frequency rTMS (1‐2 Hz) at intensities above MT increases cerebral blood flow at the stimulation site in the motor cortex (Bohning et al., 1999; Fox et al., 1997), and modifies blood flow at local and distal regions with increasingly greater modulation at higher intensities (Speer et al., 2003). In contrast, supra‐MT high‐frequency rTMS (5‐10 Hz; traditionally viewed as “excitatory”) decreases cerebral blood flow in the motor cortex (Paus et al., 1998; Siebner et al., 2000).

We did not observe significant changes in Glx concentration in the visual cortex following low‐frequency rTMS. Similarly, Stagg et al. (2009b) also report significant effects of inhibitory theta burst rTMS (cTBS) on MRS measured GABA but not Glx concentration in the motor cortex. Dose‐dependent changes in Glx concentration are, however, seen with conventional high‐frequency rTMS. A single 20‐min session of 20 Hz rTMS to the prefrontal cortex decreases Glx concentration. In contrast, single daily sessions of 20 Hz rTMS applied over five consecutive days does not affect Glx concentration at the stimulation site (prefrontal cortex), although a significant increase in Glx is observed in remote regions. These high‐frequency rTMS effects are found to be dependent on pre‐rTMS Glx levels, with lower concentrations pre‐rTMS associated with a greater increase in Glx (Michael et al., 2003).

### 4.2 Relationship between GABA+, Glx, and visual cortical excitability

We observed no significant relationship between pre‐rTMS GABA+/Glx concentrations and visual cortical excitability (PT), but a significant positive correlation between pre‐rTMS GABA+ and Glx concentrations in the visual cortex. Terhune et al. (2015) report a strong negative correlation between PT and MRS measured glutamate concentration in the visual cortex, with lower PT associated with elevated glutamate concentration, and no correlation between GABA and glutamate. Although Terhune and colleagues report concentrations as ratios relative to Cr (to normalize data), we nonetheless fail to observe these relationships in our data using the same standard reference to Cr (see Figure S1). Variability in results between studies is likely attributed to differences in TMS parameters used to obtain PT and MRS acquisition parameters. Furthermore, Terhune and colleagues measured PT following dark adaptation, which increases cortical excitability (Boroojerdi, Bushara, et al., 2000). In the motor cortex at least, a significant positive correlation is also observed between MRS measured GABA and Glx relative to Cr (Tremblay et al., 2012). Additionally, as in the visual cortex, no direct relationship is observed between MRS measured GABA and TMS measures of cortical excitability (GABA_A_ and GABA_B_ receptor activity) in the motor cortex, suggesting that overall GABA concentration may not reflect specific synaptic activity, and hence excitability levels (Stagg et al., 2011; Tremblay et al., 2012). On the other hand, TMS measures of cortical excitability, specifically GABA_B_ receptor activity, in the motor cortex are positively correlated with MRS measured glutamate concentration (Tremblay et al., 2012). GABA_B_ receptor mechanisms are instead mediated by glutamatergic activity (Chalifoux & Carter, 2011; Prout & Eisen, 1994), whereby a GABA_B_ agonist has significant excitatory rather than inhibitory effects in the visual system (Luo, Wang, Su, Wu, & Chen, 2011). Moreover, a linear relationship is suggested between neuronal glucose oxidation and glutamate/glutamine cycling (Patel et al., 2004). These findings suggest that MRS metrics of glutamate relate to synaptic glutamatergic activity (Stagg & Nitsche, 2011). MRS measures of GABA instead are considered to represent the sum of inhibitory and excitatory activity (GABA_A_ and GABA_B_ receptor activity, respectively) across all cells affected by GABAergic modulation within the VOI (Rae, 2014). Therefore, an increase/decrease in MRS measured GABA concentration does not simply imply increased/decreased inhibition, respectively. Mechanisms underlying PT are not as well understood as MT, but GABAergic mechanisms are repeatedly implicated in PT (e.g., Boroojerdi, Prager, Muellbacher, & Cohen, 2000; Mulleners, Chronicle, Vredeveld, & Koehler, 2002). The use of a GABA_A_ agonist is observed to increase PT, that is, reduce cortical hyperexcitability in the visual cortex, in migraine patients with aura (Mulleners et al., 2002). If phosphene induction via stimulation occurs via GABA_A_ receptor activity, then collectively based on prior work, neither MRS measured GABA or glutamate levels would directly be associated with PT (visual cortical excitability).

### 4.3 Effect of 1 Hz repetitive transcranial magnetic stimulation to the visual cortex on cognitive and imagery responses

Following accelerated rTMS, changes in cognitive scores measured with MoCA trended toward significance, with greater scores at 1‐week post‐rTMS compared with pre‐rTMS. Although GABA+ concentration did not show a significant change at 1‐week post‐rTMS, there was an increase relative to pre‐rTMS, and this change in cognitive effect may be related. Or simply, and more likely, the effect can be attributed to a learning effect. We employed all three MoCA versions; however, since there were four visits in the accelerated rTMS protocol, participants were tested on version 1 at pre‐rTMS and 1‐week post‐rTMS visits.

Following a single session of rTMS, VVIQ with eyes closed significantly decreased 1 hr post‐rTMS compared with pre‐rTMS, with no consistent change in GABA+/Glx concentration. It is possible that this effect is related to other mechanisms affected by a smaller dose of 1 Hz rTMS. Or perhaps, less likely, this effect may be due to mechanisms that only arise 1 hr post‐rTMS since no change was detected immediately post‐rTMS. Visual cortex activity and vividness of visual imagery show a trend for a positive correlation (Amedi, Malach, & Pascual‐Leone, 2005), and 1 Hz rTMS to the occipital cortex impairs response time of visual imagery and perceptual versions of the same tasks (Kosslyn et al., 1999). Remarkably, there was no change in VVIQ following accelerated rTMS that may be due to adaptive processes or metaplasticity (Bocci et al., 2014; Lang et al., 2004). These findings on VVIQ may have important inferences for the differential effects of single and accelerated 1 Hz rTMS sessions.

### 4.4 Therapeutic potential of accelerated 1 Hz repetitive transcranial magnetic stimulation in visual disorders

The effect of accelerated 1 Hz rTMS to the visual cortex on reducing GABA+ concentration has valuable implications for cortical plasticity. Retinal and cortical lesions trigger visual cortical plasticity necessary for reorganization and restitution of visual cortical function. Reorganization is dependent on GABAergic modulation and associated LTP that enables nearby cells to adopt topographical representation of damaged cells and continued processing of the involved region. There are major biochemical changes in adjacent deafferented synapses involving increased glutamatergic *N*‐methyl‐D‐aspartate (NMDA) and decreased GABA_A_ and GABA_B_ response in the visual cortex (Eysel et al., 1999). Similarly, in stroke, during the acute phase of recovery, peri‐infarct regions show diminished excitability associated with GABA_B_ receptor activity (Clarkson, Huang, MacIsaac, Mody, & Carmichael, 2010). In the subacute stages of stroke, downregulation of GABA increases neuronal firing (Redecker, Wang, Fritschy, & Witte, 2002), thereby also increasing NMDA function that is integral to structural (Lee, Ueno, & Yamashita, 2011) and electrophysiological reorganization, as well as reduced intracortical inhibition (Buchkremer‐Ratzmann & Witte, 1997; Liepert, Hamzei, & Weiller, 2000). Subacute GABA inhibition occurs not only in peri‐infarct regions, but also in the contralesional hemisphere, and is related to improved motor skills in the nonparetic forelimb of stroke patients (Luke, Allred, & Jones, 2004). A continued widespread reduction of GABA_A_ receptor activity, and associated excitability, persists for several months following cortical injury and is thought to facilitate plasticity changes via LTP and remapping of motor cortical representations (Carmichael, 2003; Neumann‐Haefelin, Hagemann, & Witte, 1995). This rewiring underlies at least some recovery of function seen after stroke and is still maintained in the chronic stages of stroke recovery. Previously, in occipital stroke and associated visual hallucinations (phosphenes), we have observed widespread changes in functional activity in cortical and subcortical regions, as well as altered structural connectivity between the visual cortex and frontal, parietal, and temporal regions (Rafique et al., 2016, 2018). Multiday sessions of 1 Hz rTMS to the visual cortex is capable of rebalancing functional activity across the brain following occipital stroke to a level more closely resembling controls, which corresponded to a reduction of visual hallucinations (Rafique et al., 2016). Additionally, resting‐state functional MRI data show that 1 Hz rTMS to the visual cortex modulates functional connectivity of the visual network with several cortical and subcortical nodes, including frontal, parietal, and temporal regions, as well as the thalamus, cerebellum, and brainstem (unpublished data). Stimulation of visual areas with 1 Hz rTMS also causes substantial changes in functional activity in specialized visual processing regions (Rafique, Solomon‐Harris, & Steeves, 2015; Solomon‐Harris, Rafique, & Steeves, 2016). Therefore, stimulation of the visual cortex can reach a number of distal nodes, and therefore networks, that may be differentially impaired and targeted with rTMS depending on the visual disorder in question.

Pairing rTMS treatment with vision therapies (e.g., vision restoration therapy in visual field loss) within a critical window of plasticity (e.g., 24 hr for a single day of accelerated 1 Hz rTMS) may lead to more efficient recovery and requires further investigation. Adjunct therapies can provide a better method for improving the connectivity of involved neuronal networks and for increasing synaptic efficiency, which would permit changes in neuronal excitability and rewiring to restore function with longer‐term effects (Liepert, Bauder, et al., 2000; Pekna, Pekny, & Nilsson, 2012).

The use of 1 Hz rTMS may be preferable to other noninvasive brain stimulation techniques that have differing effects on MRS measured GABA/glutamate and likely plasticity. For example, cTBS (inhibitory TBS) significantly increases MRS measured GABA in the motor cortex, with no effect on Glx concentration, which is linked to LTD‐like phenomena (Stagg et al., 2009b). Different stimulation techniques stimulate different populations or components of neurons to variable extents (e.g., Stagg Bachtiar, & Johansen‐Berg, 2011a; Stagg et al., 2009a, 2011). Further, metabolites are influenced by more than one biochemical pathway. Animal studies suggest that dopamine directly affects the excitability of GABAergic neurons, but also the modulation of dopamine occurs indirectly via GABA‐mediated activity (for a review, see Pascual‐Leone et al., 1998). These distinct effects have implications for the potential therapeutic use of stimulation paradigms where it may be clinically beneficial and more effective to use one paradigm over another. It is important to highlight that MRS quantifies the overall local concentration of metabolites and does not provide insight into changes at the synapse/receptor level (for a review, see Stagg et al., 2011b, 2011). As such, we can only speculate on the nature of changes in GABA+ concentration and consider these factors when interpreting LTD/LTP like changes.

This study provides a basic framework and preliminary results which could inform clinicians/researchers to determine appropriate use of rTMS to the visual cortex to achieve desired changes in GABA and glutamate levels at the stimulation site in patient populations. Deviations in the expected response in patient populations relative to controls can provide further valuable insight into the parameters needed for successful rTMS treatment and will implicate the underlying disease processes raising the possibility of developing biomarkers for diagnosis. Our results are from a highly screened group of healthy control participants, and effects will indeed vary in patient populations, for example, visual pathway damage, altered biochemical regulation in underlying medical conditions, interactions with medications, etc. (Maeda, Keenan, Tormos, Topka, & Pascual‐Leone, 2000; Wassermann, 2002). We also do not expect the same results with accelerated rTMS applied over consecutive days as is used in protocols for therapeutic applications (e.g., 2–5 days). Instead, we would expect an augmented response in GABA+ concentration with higher doses of accelerated rTMS based on previous research (Goldsworthy et al., 2014; Holtzheimer et al., 2010), and perhaps a dissimilar effect on Glx concentration. Similarly, the dissociative effects between single and accelerated rTMS sessions may be due to dose‐dependent effects. It may be that single sessions over consecutive days can produce a similar effect, in which case, one day of accelerated rTMS offers a more efficient protocol. Or perhaps, an opposite effect could be observed on GABA+ concentration with repeated doses of single sessions (e.g., Michael et al., 2003). The effect of single session and accelerated rTMS to the visual cortex over a greater number of days on GABA and glutamate mechanisms requires investigation. Knowledge of differential effects with a variety of protocols will enable modulation of mechanisms suited to a greater number of disorders presenting with variable pathophysiology. With longer applications of rTMS, there is greater potential for adverse effects on sensory and cognitive processing/responses, or self‐reported effects. Adverse effects should be evaluated following repeated high doses of rTMS, and due to unpredictability of effects in disease states in patient populations (Maeda et al., 2000; Wassermann, 2002). Future work requires investigation of these aspects for clinical translation of protocols relevant to visual disorders.

### 4.5 Considerations

Despite the strength of our study, several constraints should be considered. The sample size employed in this study is small; however, we minimized possible confounds (e.g., employing strict inclusion criteria to limit considerable interindividual variability) to the best of our ability, and we observe significant changes relative to a baseline measure despite a smaller sample size. The significant change in GABA+ concentration following accelerated rTMS is unlikely due to physiological fluctuations as concentrations in the occipital cortex are consistent throughout the day (Evans, McGonigle, & Edden, 2010). GABA concentration in the occipital cortex is considered stable for as long as 7 months (Near, Ho, Sandberg, Kumaragamage, & Blicher, 2014), and glutamate concentration is considered stable for at least 1 month (Henry, Lauriat, Shanahan, Renshaw, & Jensen, 2011). It is important to note that we did not control for factors such as menstrual cycle effects (Harada, Kubo, Nose, Nishitani, & Matsuda, 2011) or cycle‐linked disorders, which can cause changes in GABA receptors and neuronal function (for a review, see Bäckström et al., 2003; Epperson et al., 2002).

It could be argued that this study is somewhat limited by the absence of a sham or control stimulation site condition. Since there is extensive evidence from a broad range of studies that have addressed the efficacy of TMS versus control site/sham TMS in a variety of populations and brain regions, our study question concerned the methodological aspect of accelerated rTMS and its performance on V1 metabolites versus a single session. It is important to acknowledge that sham stimulation (whether with a sham coil or an active coil held orthogonal to the skull) presents with potential limitations as it can induce changes in neural activity via weak stimulation, clicking noises, or the tapping sensation of stimulation pulses. Sham coils can induce low strength electric fields of up to 25.3% of their respective active values (Smith & Peterchev, 2018). With the Magstim sham coil, the field under the centre of the coil is found to be minimal, whereas in an active coil the centre would have the strongest stimulation. However, the Magstim sham coil produces electric fields, and therefore stronger stimulation, in the periphery (3–7 cm from the centre). Magventure sham can increase these electric fields by up to 10% (Smith & Peterchev, 2018). Additionally, with sham rTMS, participants are aware that the stimulation sensation and clicking noise is different to active stimulation (Arana et al., 2008; Duecker & Sack, 2015; Jung, Bungert, Bowtell, & Jackson, 2016), thus unblinding participants. Despite a lack of sham condition, we nonetheless observe differential effects relative to baseline MRS measures. These different effects are observed across the two conditions relative to baseline and also relative to each condition.

It was also not feasible to include a control VOI in the same post‐rTMS MRS acquisition because of the single voxel limit. Due to MRS technical constraints, acquisition of a second VOI would not commence for ~45 min following cessation of rTMS, and immediate or shorter‐lasting measurable effects would be lost. As a result, we chose to constrain our study question to the effects of different stimulation paradigms at the stimulation site itself and remote effects of stimulation can be considered in future work.

Due to acquisition of anatomical images and MRS set‐up time (including VOI positioning and shimming), there was a delay of ~20 min between the end of rTMS and the start of MRS “ON” and “OFF” resonance acquisition that could not be avoided (MRS acquisition took an additional 13 min). Therefore, short‐lived metabolite changes, especially following single session rTMS, may not be measurable. We did not consider the effect of rTMS on other metabolites as they were not supported by our acquisition protocols. Consequently, we cannot rule out the effects of rTMS on other metabolites/biochemical pathways (Pascual‐Leone et al., 1998) that may influence our results. Moreover, rTMS effects propagate from the stimulation site to remote regions (Rafique et al., 2015; Reithler, Peters, & Sack, 2011). Because of the single voxel limit mentioned above, we cannot effectively determine remote effects of rTMS on metabolite concentrations since this would require an additional acquisition following the acquisition of the stimulation site VOI and would not capture immediate/shorter‐lasting effects. Given that we cannot accurately localize rTMS effects, the MRS measured effect of rTMS may have been dampened by the spread of rTMS to regions outside of the VOI during acquisition, or the effect may be diluted from unaffected concentrations within the VOI. Further investigation is required to address these aspects of rTMS effects prior to clinical translation.

The absence of significant rTMS effects on Glx concentration may be ascribed to the lower detection sensitivity of the MEGA‐PRESS sequence to Glx compared to GABA (Henry et al., 2011; Schubert, Gallinat, Seifert, & Rinneberg, 2004). The current sequence also cannot separate glutamate and glutamine spectra, thus, the composite value (Glx) may not accurately reflect sensitive changes in glutamate. Regardless, prior work suggests that Glx is primarily driven by the glutamate signal, as glutamate is found at ~10 times the concentration of glutamine in the brain (Stagg, 2014). There is potential for contamination of the edited signal with coedited macromolecular signals with the MEGA‐PRESS sequence. However, approaches to correct for macromolecules have their own significant limitations (Henry, Dautry, Hantraye, & Bloch, 2001; Rothman, Petroff, Behar, & Mattson, 1993), and we opted for the most common standard method to overcome macromolecule issues by reporting GABA+ concentrations. Nevertheless, variations in macromolecules between individuals are small compared with variations in GABA concentration (Hofmann, Slotboom, Boesch, & Kreis, 2001; Kreis, Slotboom, Hofmann, & Boesch, 2005; Mader et al., 2002). Therefore, it is unlikely that macromolecule contamination affects the interpretability of our observed effect in GABA+ concentration following accelerated rTMS. While GABA+ is also contaminated by homocarnosine, the in vivo concentration of homocarnosine is much lower than that of GABA (Govindaraju, Young, & Maudsley, 2000). Thus, the effect on GABA+ concentration following accelerated rTMS is also less likely to be driven by homocarnosine. GABA and glutamate concentrations do show excellent reproducibility in the visual cortex using MEGA‐PRESS (Evans et al., 2010; Henry et al., 2011; O'Gorman, Michels, Edden, Murdoch, & Martin, 2011).

Determining comparable tissue composition across visits is an indirect measure to demonstrate our consistent voxel positioning across visits. GABA concentrations are only weakly affected by voxel positioning in the occipital cortex, and as such, perfect voxel positioning is not critical (Near et al., 2014). Despite this reassurance, we remained critical in our voxel positioning to ensure consistent positioning between visits.

## 5 CONCLUSION

A single session of 1 Hz rTMS to the visual cortex does not modify GABA+ or Glx concentrations at V1. A single session would, therefore, be appropriate in settings where it is desirable to have no/minimal changes in these key neurotransmitters. A single day of accelerated 1 Hz rTMS to the visual cortex at 100% PT level significantly decreases GABA+ concentration in the visual cortex for at least 24 hr. The present approach and longer‐term investigation of accelerated rTMS effects to the visual cortex have implications for the development of treatment protocols in clinical vision applications involving altered GABAergic function. These findings have further significance for modulating visual cortical plasticity and provide insight into a critical window where adjunct therapy would be most efficient for functional restoration and recovery. The differential effects associated with single and accelerated rTMS sessions are crucial for furthering the advancement of rTMS in treatment modalities for visual‐related disorders. Future work should be directed at investigating and refining accelerated rTMS protocols on the visual cortex in patients with visual‐related disorders in order to build on this initial work.

## CONFLICT OF INTEREST

None.

## AUTHOR CONTRIBUTIONS

Sara Rafique was involved in the conceptualization, methodology, investigation, formal analysis, writing of the original draft, and funding acquisition. Jennifer Steeves was involved in the methodology, reviewing and editing, supervision, and funding acquisition.

### Peer Review

The peer review history for this article is available at https://publons.com/publon/10.1002/brb3.1845.

## Supporting information

Fig S1

## Data Availability

The data that support the findings of this study are available from the corresponding author upon reasonable request.
