# Supplementary material for: Assessing differential effects of single and accelerated low‐frequency rTMS to the visual cortex on GABA and glutamate concentrations
Source: Brain Behav. 2020 Sep 23;10(12):e01845. doi: 10.1002/brb3.1845 (PMC7749615; doi:10.1002/brb3.1845)
Supplement: Supplementary file 1 — Fig S1 [file BRB3-10-e01845-s001.pdf]

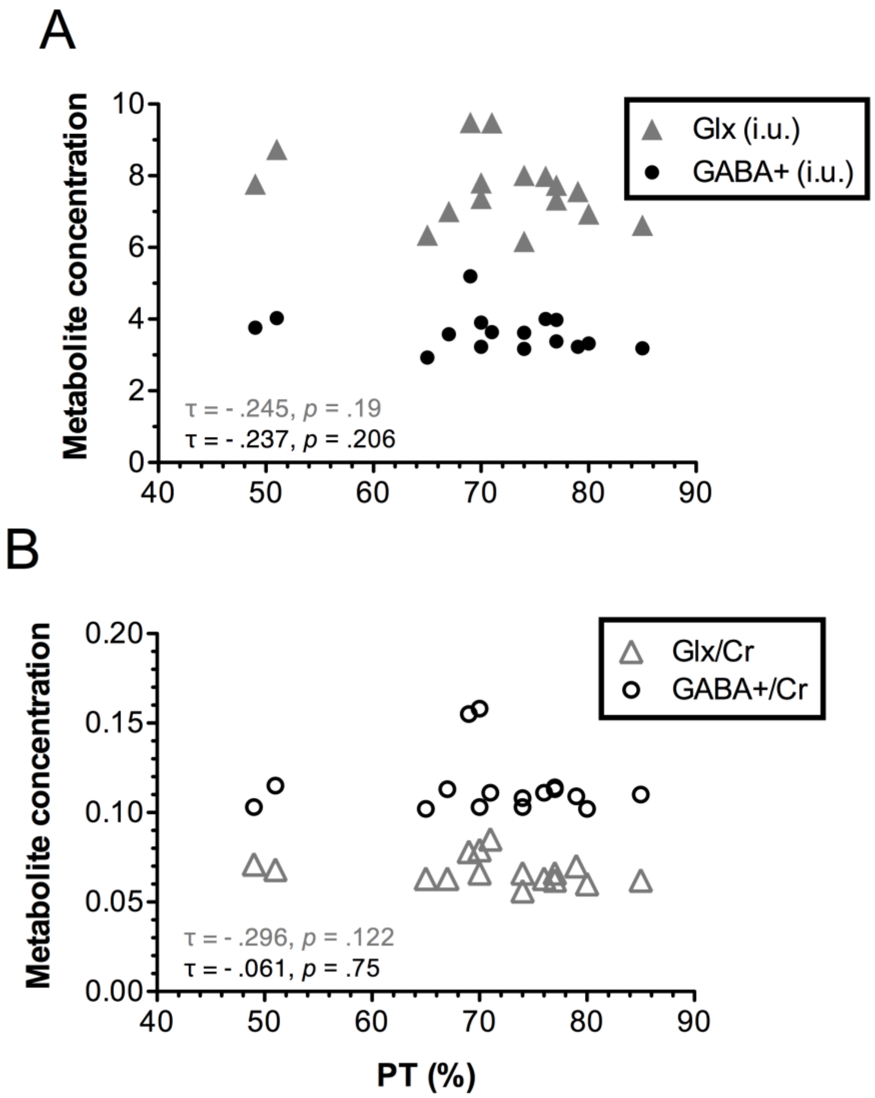


**Figure S1. No significant relationship between visual cortical excitability (phosphene threshold [PT]) and visual cortical GABA+/Glx concentrations. Related to Figure 3 and Table 1.**

Metabolites expressed as **(A)** tissue-corrected concentrations (i.u.), and **(B)** normalised concentrations using integral ratios relative to creatine (Cr). GABA+ = composite of GABA and macromolecules; Glx = glutamate and glutamine composite.
